# 12-year prediction of mild cognitive impairment aided by Alzheimer’s brain signatures at mean age 56

**DOI:** 10.1093/braincomms/fcab167

**Published:** 2021-07-23

**Authors:** McKenna E Williams, Jeremy A Elman, Linda K McEvoy, Ole A Andreassen, Anders M Dale, Graham M L Eglit, Lisa T Eyler, Christine Fennema-Notestine, Carol E Franz, Nathan A Gillespie, Donald J Hagler, Sean N Hatton, Richard L Hauger, Amy J Jak, Mark W Logue, Michael J Lyons, Ruth E McKenzie, Michael C Neale, Matthew S Panizzon, Olivia K Puckett, Chandra A Reynolds, Mark Sanderson-Cimino, Rosemary Toomey, Xin M Tu, Nathan Whitsel, Hong Xian, William S Kremen

**Affiliations:** 1 Center for Behavior Genetics of Aging, University of California San Diego, La Jolla, CA 92093, USA; 2 Joint Doctoral Program in Clinical Psychology, San Diego State University/University of California, San Diego, CA 92093, USA; 3 Department of Psychiatry, University of California San Diego, La Jolla, CA 92093, USA; 4 Department of Radiology, University of California San Diego, La Jolla, CA 92093, USA; 5 NORMENT, KG Jebsen Centre for Psychosis Research, Institute of Clinical Medicine, University of Oslo, Oslo 0316, Norway; 6 Division of Mental Health and Addiction, Oslo University Hospital, Oslo 0372, Norway; 7 Department of Neuroscience, University of California San Diego, La Jolla, CA 92093, USA; 8 Desert Pacific Mental Illness Research Education and Clinical Center, VA San Diego Healthcare System, CA 92093, USA; 9 Virginia Institute for Psychiatric and Behavior Genetics, Virginia Commonwealth University, Richmond, VA 23284, USA; 10 Center of Excellence for Stress and Mental Health (CESAMH), VA San Diego Healthcare System, San Diego, CA 92093, USA; 11 VA San Diego Healthcare System, San Diego, CA 92093, USA; 12 National Center for PTSD: Behavioral Science Division, VA Boston Healthcare System, Boston, MA 02130, USA; 13 Department of Psychiatry and the Biomedical Genetics Section, Boston University School of Medicine, Boston, MA 02118, USA; 14 Department of Biostatistics, Boston University School of Public Health, Boston, MA 02118, USA; 15 Department of Psychological and Brain Sciences, Boston University, Boston, MA 02212, USA; 16 School of Education and Social Policy, Merrimack College, North Andover, MA 01845, USA; 17 Department of Psychology, University of California Riverside, Riverside, CA 92521, USA; 18 Family Medicine and Public Health, University of California San Diego, La Jolla, CA 92093, USA; 19 Department of Biostatistics, St. Louis University, St. Louis, MO 63103, USA

**Keywords:** Alzheimer’s disease, mild cognitive impairment, mean diffusivity, cortical thickness, early prediction

## Abstract

Neuroimaging signatures based on composite scores of cortical thickness and hippocampal volume predict progression from mild cognitive impairment to Alzheimer’s disease. However, little is known about the ability of these signatures among cognitively normal adults to predict progression to mild cognitive impairment. Towards that end, a signature sensitive to microstructural changes that may predate macrostructural atrophy should be useful. We hypothesized that: (i) a validated MRI-derived Alzheimer’s disease signature based on cortical thickness and hippocampal volume in cognitively normal middle-aged adults would predict progression to mild cognitive impairment; and (ii) a novel grey matter mean diffusivity signature would be a better predictor than the thickness/volume signature. This cohort study was part of the Vietnam Era Twin Study of Aging. Concurrent analyses compared cognitively normal and mild cognitive impairment groups at each of three study waves (*n*s = 246–367). Predictive analyses included 169 cognitively normal men at baseline (age = 56.1, range = 51–60). Our previously published thickness/volume signature derived from independent data, a novel mean diffusivity signature using the same regions and weights as the thickness/volume signature, age, and an Alzheimer’s disease polygenic risk score were used to predict incident mild cognitive impairment an average of 12 years after baseline (follow-up age = 67.2, range = 61–71). Additional analyses adjusted for predicted brain age difference scores (chronological age minus predicted brain age) to determine if signatures were Alzheimer-related and not simply ageing-related. In concurrent analyses, individuals with mild cognitive impairment had higher (worse) mean diffusivity signature scores than cognitively normal participants, but thickness/volume signature scores did not differ between groups. In predictive analyses, age and polygenic risk score yielded an area under the curve of 0.74 (sensitivity = 80.00%; specificity = 65.10%). Prediction was significantly improved with addition of the mean diffusivity signature (area under the curve = 0.83; sensitivity = 85.00%; specificity = 77.85%; *P* = 0.007), but not with addition of the thickness/volume signature. A model including both signatures did not improve prediction over a model with only the mean diffusivity signature. Results held up after adjusting for predicted brain age difference scores. The novel mean diffusivity signature was limited by being yoked to the thickness/volume signature weightings. An independently derived mean diffusivity signature may thus provide even stronger prediction. The young age of the sample at baseline is particularly notable. Given that the brain signatures were examined when participants were only in their 50 s, our results suggest a promising step towards improving very early identification of Alzheimer’s disease risk and the potential value of mean diffusivity and/or multimodal brain signatures.

## Teaser

Among cognitively normal adults in their 50 s, an Alzheimer’s brain signature based on grey matter mean diffusivity—but not cortical thickness—significantly improved prediction of progression to mild cognitive impairment 12 years later. Grey matter mean diffusivity signatures may be sensitive to Alzheimer-related changes as early as middle age.

## Graphical Abstract

**Graphical Abstract:**
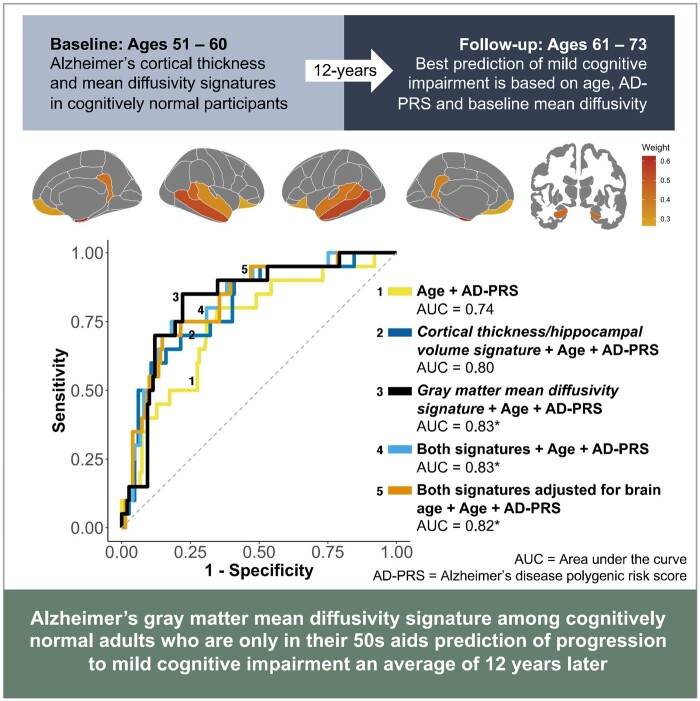

## Introduction

Neuropathological differences related to Alzheimer’s disease begin to accumulate early in the disease process,^1^ and reliable identification of these changes during the earliest stages of Alzheimer’s disease remains a critical goal. Capitalizing on regional patterns of neurodegeneration associated with Alzheimer’s disease,^2–4^ composite scores of MRI-based brain morphometry have been developed to distinguish between early Alzheimer’s disease-related atrophy and normal age-associated brain changes.^5–7^ Several of these composites, commonly termed ‘Alzheimer’s disease signatures’, have demonstrated associations with Alzheimer’s disease symptom severity and Alzheimer’s disease-related biomarkers. Additionally, they are predictive of progression to Alzheimer’s disease.^5^^,^^6^^,^^8–14^ Given that ageing is associated with atrophy in areas that partially overlap with regions used in Alzheimer’s disease signatures,^14–16^ some Alzheimer’s disease signatures have been adjusted for predicted brain age in an effort to improve their predictive utility.^16^

The majority of research on Alzheimer’s disease signatures has involved adults over 70 years of age.^5^^,^^6^^,^^8–14^ A rare study examined prediction of progression to MCI rather than Alzheimer’s disease based on a cortical thickness Alzheimer’s disease signature in cognitively normal (CN) middle-aged adults.^7^ Lower cortical thickness signature scores (indicating thinner cortex) were associated with increased risk of progression to MCI within 7 years of baseline (average age = 56 years, SD = 10.4), but not with progression that occurred more than 7 years from baseline. Participants were highly educated (mean = 17.0 years), 75% had a first-degree relative with Alzheimer’s disease, and the age range was relatively wide. Almost all prior research has also been limited to Alzheimer’s disease signatures that rely on macrostructural neuroimaging methods that are unable to detect microstructural changes that may present early in the disease process. To our knowledge, a single exception is a cross-sectional study using a composite based on multi-shell diffusion measures [neurite density and orientation dispersion (NODDI)], which reflect neural microstructure.^17^ Their diffusion-based signature discriminated between CN and MCI participants. Here again, the sample was older (mean age = 73 years), highly educated (mean = 15.8 years), and at elevated risk for Alzheimer’s disease (47.6% *APOE*-ε4-positive). Additional research is thus warranted to determine the utility of different Alzheimer’s disease signatures to predict MCI/Alzheimer’s disease in middle-aged samples that are also more representative in terms of education and Alzheimer’s disease risk.

Alzheimer’s disease signatures derived from diffusion MRI (dMRI), such as grey matter mean diffusivity (MD), may be a particularly useful early Alzheimer’s disease-related biomarker. Although most dMRI studies have focussed on white matter, we examined grey matter because of its greater relevance for Alzheimer’s disease. In grey matter, variation in MD may reflect microstructural integrity of neurites and cell bodies by measuring the average water diffusion within a voxel, which increases as microstructural barriers degenerate.^18^ Some evidence suggests that these microstructural changes may predate macrostructural atrophy as measured using conventional structural MRI techniques.^18^^,^^19^ Several studies have found increased cortical MD in individuals with MCI or Alzheimer’s disease.^18^^,^^20–23^ Our group has shown that variation in cortical and subcortical grey matter MD is heritable and partly influenced by genetic factors that are distinct from genetic factors influencing cortical thickness or subcortical volumes.^24^^,^^25^ These findings suggest that measures of cortical MD may capture information regarding neuropathological changes early in the disease process distinct from those measured using cortical thickness or subcortical volumes.

Our group previously used data from the Alzheimer’s Disease Neuroimaging Initiative (ADNI; average age = 75.7 years, SD = 5.0) to identify eight cortical regions of interest (ROIs) that discriminated between CN older adults and individuals with mild Alzheimer’s disease^9^ and predicted progression from MCI to Alzheimer’s disease.^11^ This signature of cortical thickness and hippocampal volume (referred to as ‘Alzheimer’s disease thickness/volume signature’) was unique in that it used ROI-specific weights to appropriately reflect the differential rates of atrophy across regions observed in Alzheimer’s disease.^6^ Here, we investigated the ability of this published thickness/volume signature and a novel grey matter MD composite using the same weights and regions (referred to as ‘MD signature’) to predict incident MCI over an average follow-up of 12 years among a nationally representative cohort of men in their 50 s. We used the same weightings for the MD signature because an MD signature has not yet been developed and validated in a sample of Alzheimer’s disease cases and controls. We specifically tested whether these Alzheimer’s disease signatures improved longitudinal predictions over and above two established risk factors: age^26–29^ and Alzheimer’s disease polygenic risk.^30–33^ We hypothesized that: (i) Alzheimer’s disease signatures in CN middle-aged adults would predict progression to MCI; and (ii) the MD signature would be a better predictor than the thickness/volume signature because it is more sensitive to earlier brain changes. Additional complementary analyses examined concurrent signature score differences in MCI and CN participants. We also performed analyses adjusting for predicted brain age difference scores to differentiate between general brain ageing and more specific Alzheimer’s disease-related brain changes.

## Materials and methods

### Participants

Participants were men from the Vietnam Era Twin Study of Aging (VETSA), a longitudinal study beginning in middle age.^34–36^ They comprise a nationally representative, community-dwelling sample of male–male twins who are similar to American men in their age range with respect to health and lifestyle characteristics.^37^ At baseline, all participants were between the ages of 51 and 60 years. All served in the US military at some point between 1965 and 1975. Approximately 80% reported no combat exposure. For the present analyses, exclusionary criteria included conditions that could contribute to cognitive impairment unrelated to MCI: seizure disorder, multiple sclerosis, stroke, HIV/AIDS, schizophrenia or substance dependence.^38^ Traumatic brain injury was not an exclusion criterion because it is associated with increased risk for MCI; however, the CN and MCI groups did not differ with respect to traumatic brain injury.

Table 1 shows the sample characteristics. We examined concurrent group differences at the three study waves (*n *=* *564; Table 1) between individuals who remained CN at all waves for which they had data (robust-CN, *n *=* *489) and individuals diagnosed with MCI (*n *=* *75). For these analyses, participants were excluded if they reverted from MCI to CN at any subsequent wave.

**Table 1:** Concurrent analyses: Demographic characteristics of cognitively normal men and those with MCI at each wave.

	All in analyses (*n* = 564)	Wave 1		Wave 2		Wave 3	
		Robust-CN	MCI	Robust-CN	MCI	Robust-CN	MCI
Sample size with thickness/vol signature	561	339	28	276	29	288	48
Sample size with MD signature	497	258	22	245	26	209	37
Age range	51–71	51–60	51–60	55–65	55–65	61–71	61–71
Age	61.68 (5.36)	56.10 (2.60)	56.93 (2.53)	61.83 (2.64)	62.44 (2.43)	67.72 (2.57)	67.40 (2.66)
Years of education	13.88 (2.16)	14.00 (2.23)^*^	12.86 (1.60)^*^	13.92 (2.14)	13.38 (1.70)	14.24 (2.14)	13.58 (2.13)
AD-PRS	58.38 (7.52)	58.38 (7.61)	59.26 (6.29)	57.65 (7.12)	57.56 (6.76)	57.77 (7.58)^*^	60.70 (8.41)^*^
Number of *APOE*-ε4 carriers (%)	133/564 (24%)	89/339 (26%)	6/28 (21%)	69/276 (25%)	7/29 (24%)	63/288 (22%)	12/48 (25%)

Mean (SD) descriptive statistics by group (robust-CN and MCI) at each wave. For Waves 1–3, demographic characteristics of sample with thickness/volume signature are reported; characteristics of sample with MD signature did not differ from sample with thickness/volume signature at any wave (*P*s > 0.05). Three participants with data for the MD signature did not have useable data for the thickness/volume signature at any wave. The VETSA sample comprises participants involved in multiple waves, and some entered the study after wave 1. Additional information about sample sizes across waves is available in the supplemental materials.

AD-PRS, Alzheimer’s disease polygenic risk score; CN, cognitively normal; MCI, mild cognitive impairment; MD, mean diffusivity.

* *P* < 0.05 for differences between robust-CN and MCI groups at a particular wave.

Predictive analyses included 169 participants who met the following criteria: (i) CN at baseline; (ii) had both Alzheimer’s disease signature scores at baseline and an Alzheimer’s disease polygenic risk score (AD-PRS); and (iii) had cognitive data at Wave 3 (Table 2). At the Wave 3 follow-up, 20 (11.83%) converted to MCI. The study was approved by the Institutional Review Boards at the University of California, San Diego (UCSD), Boston University, and the Massachusetts General Hospital (MGH).

**Table 2:** Predictive analyses: characteristics of individuals who remained cognitively normal and those who progressed to MCI by Wave 3

	Remained CN	Progressed to MCI
*N*	149	20
Age at baseline	55.90 (2.66)	57.17 (2.16)
Age at Wave 3	67.02 (2.71)	68.45 (2.19)
Years of education	14.09 (2.36)	14.35 (1.95)
AD-PRS	58.59 (8.03)	60.71 (7.33)
Number of *APOE-*ε4 carriers (%)	39/149 (26%)	7/20 (35%)
Baseline thickness/volume signature *z* score	−0.03 (1.01)	0.21 (0.90)
Baseline MD signature *z* score	−0.03 (1.02)	0.25 (0.83)

Mean (SD) demographic characteristics by group (remained CN or progressed to MCI at Wave 3). All participants were CN at Wave 1. All group comparisons were nonsignificant (*P*s > 0.05).

CN, cognitively normal; MCI, mild cognitive impairment; MD, mean diffusivity.

### Definition of mild cognitive impairment

The Jak–Bondi approach was used to diagnose MCI.^39^^,^^40^ In a direct comparison of ADNI participants with diagnoses based on Petersen criteria, Jak–Bondi diagnoses were associated with a higher proportion of participants progressing to Alzheimer’s disease, a lower proportion reverting to normal, a higher proportion being Alzheimer’s disease biomarker-positive, and a higher proportion being *APOE*-ε4 positive.^41^

Participants completed a neuropsychological battery comprising 18 tests that encompassed six cognitive domains: memory, executive functioning, attention, language, visuospatial ability and processing speed.^38^ Criteria for impairment within a domain required performance on 2+ tests that were each >1.5 SDs below age- and education-adjusted normative means. Although the Jak–Bondi approach allows for different impairment cut-offs,^40^ requiring performance >1 SD below normative means on 2+ tests is most common. However, in prior work with Drs. Jak and Bondi, we used the more conservative threshold of 1.5 SDs because of the younger age of the VETSA sample.^42^ The more commonly used threshold identified an unrealistic 32% of this relatively young sample as having MCI. In line with psychometric principles, we determined that the threshold needed to be more conservative given the expected lower base rate of MCI in a sample as young as the VETSA cohort.

To account for longstanding differences in cognitive performance, all scores were adjusted for a measure of general cognitive ability that was previously administered to participants at an average age of 20 years.^43^ Scores for returning participants from Waves 2 and 3 were additionally adjusted for practice and attrition effects using a replacement-subjects method as described previously.^44^^,^^45^ Briefly, this method assumes that if two groups are drawn from the same population and the only difference is that one group has taken a test before and the other is being tested for the first time, any group difference can be attributed to practice effects. Therefore, we compared the scores between returnees taking the test for a second time and the scores of demographically-matched attrition replacement subjects taking the test for the first time. To avoid overestimating practice effects because dropouts are usually lower functioning than returnees, this approach also calculates the attrition effect as the difference in the mean score of returnees at a prior wave and the mean score of all individuals at that prior wave. The practice effect of each test was then calculated as the difference score minus the attrition effect.^45^

### MRI acquisition and processing

Images at Wave 1 (baseline) were acquired on Siemens 1.5 T scanners at UCSD and MGH. Images at Wave 2 were acquired with a GE 3 T Discovery 750x scanner (GE Healthcare, Waukesha, WI, USA) with an 8-channel phased array head coil at UCSD and with a Siemens Tim Trio (Siemens USA, Washington, D.C.) with a 32-channel head coil at MGH. Images at Wave 3 were acquired at UCSD with two GE 3 T Discovery 750× scanners with eight-channel phased array head coils. Volumetric segmentation^46^^,^^47^ and cortical surface reconstruction^46–49^ methods were performed with FreeSurfer version 5.1 (http://surfer.nmr.mgh.harvard.edu Accessed 30 July 2021). Raw images were visually inspected for quality and excluded for excessive motion or acquisition artefacts. Processed structural images were manually edited to correct errors in cortical surface reconstruction and subcortical segmentation was visually reviewed. Subjects with major segmentation or surface reconstruction failures were excluded from analysis.

Structural MR images were processed as described previously.^46–51^ Briefly, this involves correction of distortion due to gradient nonlinearity,^52^ image intensity normalization,^53^ rigid registration into standard orientation with 1 mm isotropic voxel size, and removal of non-brain tissue. Boundaries between grey matter, white matter, and CSF were defined and the cortical surface was then divided into 66 distinct regions (33 per hemisphere) according to the Desikan-Killiany atlas.^47^^,^^54^ Mean cortical thickness was calculated for each ROI.

Briefly, diffusion MRI images were processed as follows. A robust and accurate procedure for reducing spatial and intensity distortions in EPI images caused by B0 field was applied^55^ that relies on the reversing gradient method.^56^^,^^57^ Pairs of *b* = 0 (i.e. non-diffusion weighted) images with opposite phase encoding polarities (and thus opposite spatial and intensity distortion patterns) were aligned using a fast, nonlinear registration procedure. The displacement field volume was adjusted (i.e. translation and rotation) for estimated head motion and then used to unwarp distortions in each frame (i.e. diffusion-weighted volume). Eddy current distortions were corrected with a nonlinear estimation procedure that used the diffusion gradient orientations and amplitudes to predict the pattern of distortions across the entire set of diffusion weighted volumes.^58^ Corrections were restricted to translation and scaling along the phase-encode direction, avoiding spurious head rotations introduced by affine registration. Gradient nonlinearity distortions were corrected for each frame.^52^ Head motion was corrected by registering each frame to a corresponding image synthesized from a tensor fit, thus accounting for variation in image contrast across diffusion orientations.^59^ The diffusion gradient matrix was then adjusted for head rotation.^59^^,^^60^ For both motion correction and eddy current distortion correction, robust diffusion tensor estimation was used to exclude the contribution of dark slices, which are caused by abrupt, severe head motion. As part of motion correction, such outlier values were replaced—an entire slice at a time for a given diffusion direction—with values synthesized from the robust tensor fit. T_2_-weighted *b* = 0 images were registered to T_1_-weighted structural images using mutual information^61^ after coarse pre-alignment via within-modality registration to atlas brains. This provides a robust and accurate registration, and registrations were reviewed manually for quality. Diffusion-weighted images were resampled into a standard orientation with 2 mm isotropic resolution; the number of resampling steps was reduced by combining this with the motion correction. Cubic interpolation was used for all resampling steps. Conventional diffusion tensor imaging (DTI) methods were used to model the diffusion tensor as an ellipsoid where eigenvalues λ1, λ2 and λ3 define the three primary axes,^62–64^ and MD was calculated as the average diffusion in all directions. Raw and processed images were visually inspected to exclude data with severe scanner artefacts or excessive head motion.

At each vertex, seven samples were taken in 0.2 mm increments along the vector normal to the grey/white boundary surface, from 0.8 to 2 mm outwards into the cortical mantle. Multiple samples were taken because these vectors may be oblique to the image matrix, and can thus pass through multiple voxels with varying properties. Samples were not taken at the boundaries of the cortical ribbon due to the potential for small errors in co-registration which may result in edges partially overlapping with CSF or white matter. In order to minimize the effects of partial voluming and regional variations in cortical thickness, we calculated weighted averages of MD based on the proportion of grey matter in each voxel. White and grey matter voxels were labelled using the cortical surfaces generated by FreeSurfer in processed T_1_ image resolution (1 mm isotropic). These masks were resampled into the resolution of the pre-processed dMRI image (2 mm isotropic) using cubic interpolation. Thus, every voxel in the dMRI image was given a corresponding volume fraction ranging from 0 to 1 representing the proportion of each tissue type contained in the voxel. The 7 samples along the normal vector at each surface vertex were averaged using weights determined by the grey matter volume fraction map. The weighting factor given to each voxel was calculated using Tukey's bisquare weight function^65^:

wx= (1-(1-vt)2)2,v>t\0,v≤t

where *v* is the volume fraction and *t* is a tunable scaling factor, here set to 0.5. In practical terms, this will set any voxel with a volume fraction less than 0.5 to 0, and any voxel with a volume fraction above 0.5 to a weight ranging between 0 and 1. This weighting function was used to exclude or downweight the contribution of voxels with a low proportion of grey matter. In addition to its use in robust estimation, previous uses of the Tukey bisquare weight function have included edge-finding in noisy images,^66^ image registration^67^ and image segmentation.^68^ Average grey matter MD was calculated for all 66 ROIs in the Desikan–Killiany atlas.^54^

For subcortical ROIs, contamination due to partial voluming in the ROI with CSF is suppressed by calculating weighted averages. Specifically, weighting factors for each voxel in the ROI are calculated based on the difference of MD values relative to the median within each ROI. The typical dispersion of MD values is defined for each ROI as the median absolute deviation from the median (MAD), averaged across subjects. Weighting factors are calculated using Tukey’s bisquare function such that lower weights are assigned to voxels with MD values farther from the median value, relative to the dispersion values multiplied by 4.7.

### Alzheimer’s disease polygenic risk score

Summary data from the Alzheimer’s disease genome-wide association study (GWAS) of European-descent subjects^69^ were used to compute the AD-PRS for VETSA participants in a prior publication.^33^ We included the first 3 principal components as covariates to account for any cryptic population substructure.^33^ As polygenic risk scores are not as predictive outside of the population used to calculate the GWAS,^70^ and because VETSA has so few non-European subjects, AD-PRSs were calculated only for non-Hispanic white participants of European-American ancestry.^33^ In the VETSA sample, higher AD-PRSs have been associated with significantly greater odds of having MCI than being CN.^33^ Genotyping methods have been described previously.^33^

### Alzheimer’s disease brain signatures and predicted brain age difference scores

**Figure 1:**
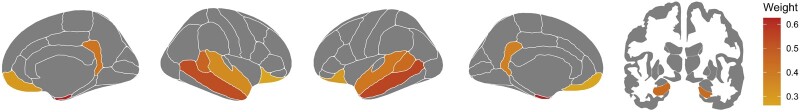
**Regions and corresponding weights used to create Alzheimer’s disease signatures.** The cortical thickness/volume signature is a weighted average of thickness in seven cortical regions (entorhinal cortex, middle temporal gyrus, bank of superior temporal sulcus, superior temporal gyrus, isthmus cingulate, lateral orbitofrontal cortex and medial orbitofrontal cortex) plus hippocampal volume, with separate weights for left and right hemisphere regions. We applied the same weightings to MD values for each of these ROIs to generate our novel MD signature scores.

First, we used an Alzheimer’s disease brain signature previously developed by our group using independent data from ADNI.^9^^,^^11^ This signature is a weighted average of thickness in seven cortical regions plus hippocampal volume, with separate weights for left and right hemisphere regions: entorhinal cortex (LH = 0.626; RH = 0.586), middle temporal gyrus (LH = 0.554; RH = 0.496), bank of superior temporal sulcus (LH = 0.444; RH = 0.435), superior temporal gyrus (LH = 0.410; RH = 0.322), isthmus cingulate (LH = 0.377; RH = 0.387), lateral orbitofrontal cortex (LH = 0.303; RH = 0.245), medial orbitofrontal cortex (LH = 0.263; RH = 0.279) and hippocampus (LH = 0.696; RH = 0.621). Figure 1 displays the regions and weights used to create the signatures. We regressed out effects of age and scanner for each ROI, as well as estimated intracranial volume for the hippocampus. Standardized residuals of ROIs were then weighted accordingly and summed together to form the thickness/volume signature scores. Second, because there is as yet no independently created Alzheimer’s disease grey matter MD signature, we applied these same weightings to the MD values for each ROI and carried out the same steps to generate our novel MD signature scores.

To account for variance associated with general ageing, we also examined thickness/volume and MD signature scores after adjusting for predicted brain age difference (PBAD) scores. In doing so, we sought to determine if removing variance associated with general ageing would improve the Alzheimer’s disease signatures by presumably making them more Alzheimer’s disease-specific, or if the signatures were really just functioning as indices of general ageing. A similar approach has been applied to the Alzheimer’s disease brain signature of Dickerson et al.^5^ in older ADNI participants, and adjusting for age-related variance increased the predictive utility of their cortical thickness Alzheimer’s disease signature.^16^ Predicted brain age was calculated using the publicly available Brain-Age Regression Analysis and Computation Utility Software (BARACUS).^71^ Detailed PBAD methodology has been described elsewhere by our group.^72^ PBAD was calculated by subtracting predicted brain age from chronological age, such that lower (more negative) values indicate more advanced brain age.

### Statistical analysis

#### Concurrent group differences

Concurrent group differences in signature scores between robust-CN and MCI were examined using linear mixed effects models. Subjects nested within twin pairs were included as random effects to control for repeated measures within subject across the waves and correlations within twin dyads. Group (robust-CN, MCI), wave (1, 2, 3), and their interaction were included as fixed effects. Significant associations were determined using type III fixed effects with Satterthwaite’s approximation method. Effect sizes were calculated using a standardized mean difference (SMD) measure analogous to Cohen’s *d*.^73^^,^^74^

### Predictive analyses

In separate predictive analyses, we investigated the ability of signatures to predict CN versus MCI at the Wave 3 follow-up. We fit a series of mixed effects logistic regression models with the following predictors:

Model 1: age + AD-PRS

Model 2: age + AD-PRS + thickness/volume signature

Model 3: age + AD-PRS + MD signature

Model 4: age + AD-PRS + thickness/volume signature + MD signature

Model 5: age + AD-PRS + PBAD-adjusted thickness/volume signature + PBAD-adjusted MD signature.

All predictors were *z*-scored and all models included a random effect to account for non- independence of observations in the twin data. Area under the curve (AUC) of receiver operating characteristics (ROCs) between models were statistically compared using a stratified bootstrap method with 2000 permutations using predicted probabilities from fixed effects estimates of the mixed models. Optimal ROC thresholds were calculated using Youden’s J statistic, which maximizes the sum of sensitivity and specificity.^75^ A false discovery rate of 0.05 was applied separately to concurrent and predictive analyses to control for multiple comparisons;^76^ original *P-*values are reported. All analyses were conducted using R v4.0.0 in RStudio v1.2.1335.^77–80^

### Data availability

VETSA data are publicly available to qualified researchers, with restrictions. Information regarding data access can be found at http://www.vetsatwins.org/for-researchers/.

## Results

### Concurrent group differences

**Figure 2:**
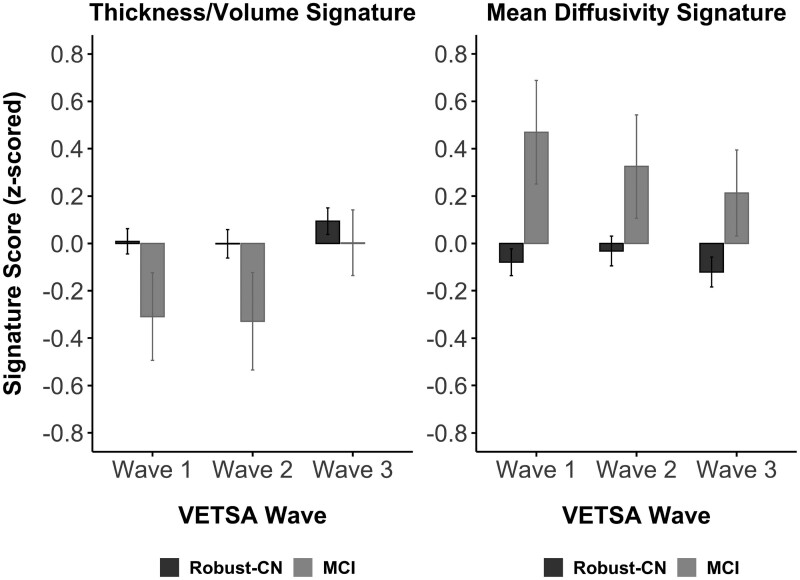
**Concurrent group differences in signature scores.** Mean standardized signature scores by group (robust-CN or MCI) at each VETSA wave. Error bars represent ±1 standard error of the mean. In linear mixed effects models, no significant group differences were observed in thickness/volume signature scores (*t*_603.03_ = 1.85, *P* = 0.065, *n* = 561, SMD = 0.16). Individuals with MCI displayed significantly higher MD signature scores compared to the robust-CN group across the three VETSA waves (*t*_527.15_ = 3.99, *P* < .001, *n* = 497, SMD = 0.36).

Figure 2 displays mean standardized signature scores by group at each study wave. Linear mixed effects models revealed nonsignificant group-by wave interactions for both thickness/volume [*F*(2, 718.68) = 0.888, *P *=* *0.412] and MD signature scores [*F*(2, 636.20) = 0.010, *P *=* *0.990], indicating that the concurrent associations between signature scores and MCI status were not different across waves. Accordingly, models were rerun with only the main effects of group and wave as fixed effects. The effect for thickness/volume signature scores was in the expected direction with the MCI group demonstrating lower scores compared to the robust-CN group, but the difference was only at trend level (*t*_603.03_=1.85, *P *=* *0.065, SMD = 0.16). In contrast, the MCI group showed significantly higher (worse) MD signature scores compared to the robust-CN group (*t*_527.15_=3.99, *P *<* *0.001, SMD = 0.36). Similar results were obtained after adjusting signature scores for PBAD, such that group differences were observed for PBAD-adjusted MD signature scores (*t*_550.67_=3.60, *P*<0.001, SMD = 0.45) but not for PBAD-adjusted thickness/volume signature scores (*t*_599.20_=1.40, *P *=* *0.162, SMD = 0.16). The correlation between thickness/volume and MD signature scores was small at Wave 1 (*r=* −0.28, *P *<* *0.001), medium at Wave 2 (*r=* −0.50, *P *<* *0.001), and large at Wave 3 (*r=* −0.62, *P *<* *0.001). All significant effects survived correction for multiple comparisons.

### Prediction of MCI based on baseline Alzheimer’s disease signatures

**Figure 3:**
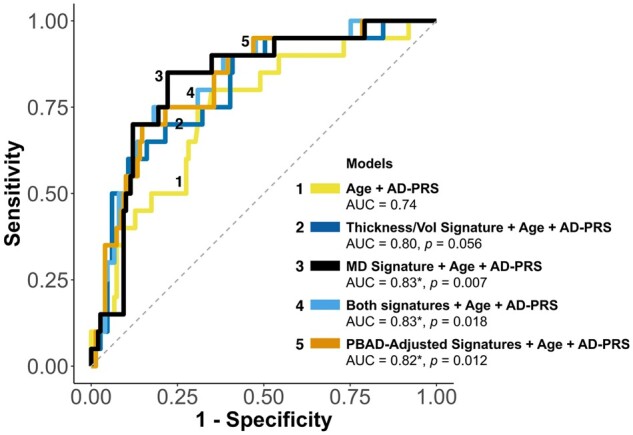
**Predicting 12-year progression to MCI: ROC curves.** (*) indicates a significantly higher AUC compared to a model with age and AD-PRS alone after FDR correction; unadjusted *p* values for each model comparison are displayed in the figure. The same 169 participants were included in all models. Dotted line representing AUC = 0.50 is displayed for reference. AD-PRS, Alzheimer’s disease polygenic risk score; MCI, mild cognitive impairment; MD, mean diffusivity; PBAD, predicted brain age difference; ROC, receiver operating characteristics; Vol, volume.

Table 3 shows the results of the regression models using baseline data to predict MCI status 12 years later at Wave 3. ROC curves are displayed in Figure 3. Our model with only age and AD-PRS (Model 1) yielded an AUC of 0.74 (95% CI: 0.61–0.85; sensitivity = 80.00%; specificity = 65.10%; accuracy = 66.86%). Adding the thickness/volume signature (Model 2) resulted in a numerically higher AUC of 0.80 (95% CI: 0.70–0.89; sensitivity = 60.00%; specificity = 89.26%; accuracy = 85.80%), though this model was not a significant improvement over Model 1 (*P *=* *0.056). In contrast, adding the MD signature to Model 1 (Model 3) did result in a significant improvement in model fit (AUC = 0.83; 95% CI: 0.73–0.91; sensitivity = 85.00%; specificity = 77.85%; accuracy = 78.70%; *P *=* *0.007).

**Table 3:** Predicting 12-year progression to MCI: ROC results

Model	AUC	Sensitivity	Specificity	Accuracy	PPV	NPV
1. Age + AD-PRS	0.74	0.80	0.65	0.67	0.24	0.96
2. Thickness/Vol + Age + AD-PRS	0.80	0.60	0.90	0.86	0.43	0.94
3. MD + Age + AD-PRS^a^	0.83	0.85	0.78	0.79	0.34	0.97
4. Thickness/Vol + MD + Age + AD-PRS^a^	0.83	0.75	0.82	0.81	0.36	0.96
5. PBAD-Adjusted Signatures + Age + AD-PRS^a^	0.82	0.70	0.85	0.83	0.39	0.95

Optimal threshold was selected for high and balanced sensitivity and specificity (Youden method).

AD-PRS, Alzheimer’s disease polygenic risk score; AUC, area under the curve; MCI, mild cognitive impairment; MD, mean diffusivity; ROC, receiver operating characteristics; PPV, positive predictive value; NPV, negative predictive value; PBAD, predicted brain age difference; Vol, volume.

a A model with significantly higher AUC compared to a model with Age + AD-PRS after FDR correction.

A model (Model 4) that included both signatures, age, and AD-PRS also significantly improved the AUC over Model 1 (AUC = 0.83; 95% CI: 0.73–0.91; sensitivity = 75.00%; specificity = 81.88%; accuracy = 81.07%; *P *=* *0.018), but did not appreciably differ from Model 3. Adjusting the two signatures for PBAD in Model 5 did not substantively change the predictive value (AUC = 0.82; 95% CI: 0.72–0.91; sensitivity = 70.00%; specificity = 85.23%; accuracy = 83.43%). Secondary analyses that replaced the AD-PRS with *APOE* status (ε4+ versus ε4−) in models 1–5 yielded significantly lower AUCs, suggesting that the predictive utility of the AD-PRS was not driven solely by *APOE* (Supplementary Table 1).

Odds ratios (ORs) from Model 4 indicated a positive association among all predictors and incident MCI: thickness/volume signature OR = 1.65 (95% CI: 0.78–3.60; *P *=* *0.265); MD signature OR = 2.23 (95% CI: 0.96–5.67; *P *=* *0.089); age OR = 2.92 (95% CI: 1.12–6.37; *P *=* *0.057); and AD-PRS OR = 1.69 (95% CI: 0.79, 3.29; *P *=* *0.235). The direction of effect for the thickness/volume signature was unexpected because greater thickness/volume at Wave 1 was associated with higher odds of MCI at Wave 3. Given this counterintuitive finding, we examined the signatures across age for individuals who were CN at Wave 1 and remained CN (CN-stable) or who progressed to MCI by Wave 3 (CN-MCI) in post hoc analyses using mixed models that accounted for non-independence of twins and repeated measurements across waves. The CN-MCI group displayed slightly higher thickness/volume signature scores at earlier ages, but lower thickness/volume signature scores at later ages (roughly after age 65) compared to the CN-stable group (Supplementary Figure 1a). In contrast, the MD signature scores trended higher (worse) across age for the CN-MCI group compared to the CN-stable group (Supplementary Figure 1b).

## Discussion

We demonstrated that MRI-derived Alzheimer’s disease signatures among CN adults who are only in their 50 s can aid in prediction of progression to MCI an average of 12 years later. Adding the MD signature alone significantly improved AUC values over age and AD-PRS, but additionally including the thickness/volume score added little predictive utility. Given that the majority of research concerning Alzheimer’s disease signatures has involved adults over 70 years of age,^5^^,^^6^^,^^8–14^ the finding that an Alzheimer’s disease signature in middle-aged adults can improve prediction of progression to MCI over a decade later has important implications for very early identification of individuals at risk for MCI or Alzheimer’s disease. Additionally, the finding that adjusting signatures for PBAD did not substantively change predictions lends support to the idea that these signatures are Alzheimer’s disease-related and not just indicators of general ageing.

The predictive utility of these MRI-based signatures, when combined with age and a static marker of Alzheimer’s disease risk (AD-PRS), appears to be reasonably comparable to findings using other biomarkers of Alzheimer’s disease pathology among older adults over shorter intervals. We do not see Alzheimer’s disease signatures as sufficient by themselves; rather, they represent an additional biomarker that might improve prediction. For example, Steenland et al.^81^ demonstrated that total tau and amyloid-β_42_ together predicted progression from CN to MCI over a 3-year follow-up period (average baseline age = 74.5, SD = 5.5) with 68% sensitivity, 64% specificity, and 65% accuracy. In a sample of similar mean baseline age as the present study but with a much larger age range (average baseline age = 56.9, SD = 8.4), CSF amyloid-β and phosphorylated tau together with age and education predicted progression from CN to MCI over a 5-year follow-up with 57% sensitivity and 75% specificity.^82^ The same study found a combination of eight measures (age, education, amyloid-β, phosphorylated tau, hippocampal and entorhinal cortex volume, Digit Symbol Substitution test and Paired Associates Immediate Recall scores, and *APOE*-ε4 status) best predicted progression from CN to MCI over a 5-year follow-up period with 80% sensitivity and 75% specificity. Over a 10-year follow-up, these eight measures predicted progression from CN to MCI with 74% sensitivity and 77% specificity. Notably, participants were highly educated (mean = 17.1 years), the majority had a family history of dementia, and predictive ability was based on data derived within the same sample, which may bias diagnostic accuracy. Our MD signature was composed of ROIs and weights derived in an independent sample. Together with age and AD-PRS, it predicted incident MCI over a 12-year follow-up period with 85% sensitivity, 78% specificity, and 79% accuracy.

There is also an extensive literature demonstrating the predictive utility of standard Alzheimer’s disease biomarkers (*e.g.* amyloid-β_42_, tau, neurofilament light chain) among CN older adults for progression to MCI or Alzheimer’s disease.^83–88^ Many of these studies report significantly increased hazard ratios or progression rates for clinical decline among CN individuals with elevated or abnormal Alzheimer’s disease biomarkers. However, direct comparison to our study is limited due to differences in outcome measures and sample characteristics. Many of these studies included Alzheimer’s disease as an outcome in addition to MCI, most involved adults over the age of 70 at baseline, and some were based on CN individuals who reported subjective cognitive decline at baseline. The baseline age difference is particularly important given our focus on early identification. Our baseline age is generally 15–20 years younger than in most of these other studies, and our average outcome age is younger than the average baseline age of most of the other studies. Future work is likely to benefit from examining the predictive utility of MRI-based Alzheimer’s disease signatures in combination with biomarkers of Alzheimer’s disease pathology among middle-aged adults. Although it is well known that age is the best predictor of Alzheimer’s disease and other dementias,^26–29^ that is true when considering a relatively wide age range. The predictive utility of age was limited in the VETSA sample due to the narrow age range (51–60 years at baseline). We cannot rely on age as a strong predictor if the goal is to be able to predict disease risk at a relatively young age. Our results, however, suggest that combining age, polygenic risk, and Alzheimer’s disease brain signatures may bring us closer to such prediction.

Model 3 with the MD signature, age, and AD-PRS was associated with greater sensitivity (0.85; 17/20 MCI cases correctly classified) compared to Model 2 with the thickness/volume signature, age, and AD-PRS (0.60; 12/20 MCI cases correctly classified). Interestingly, the thickness/volume signature showed a greater degree of specificity (0.90; 134/149 CN cases correctly classified) compared to the MD signature (0.78; 118/149 CN cases correctly classified). This difference may suggest that each signature captures some unique information, yet combining them did not improve prediction over the MD signature alone. A related cross-sectional study by Vogt et al.^17^ using NODDI also found that neural microstructure measures did better at discriminating between CN and MCI participants than measures of cortical thickness (AUC = 0.71 versus 0.66, respectively), though combining measures in that study also did not substantially improve discrimination (AUC = 0.72). Diagnostic accuracy in the Vogt et al. study was determined using ROI-based results from within the same study. Our AUC of 0.83 was obtained with much younger participants and, as noted above, with Alzheimer’s disease signatures that were derived from an independent sample.

Unexpectedly, we found the baseline thickness/volume signature was positively (albeit nonsignificantly) associated with incident MCI (OR = 1.65). Post-hoc examination suggested that the thickness/volume signature was greater among the CN-MCI group compared to the CN-stable group at younger ages, but this effect reversed at later ages, presumably with the onset of MCI (Supplementary Figure 1a). Although this finding seems counterintuitive, previous cross-sectional studies have also found increased cortical thickness among amyloid-positive CN individuals compared to CN individuals without evidence of Alzheimer’s disease pathology, but decreased cortical thickness among individuals who had already progressed to MCI or Alzheimer’s disease.^89^^,^^90^ It has been suggested that this may represent an inverted-U pattern in which cortical thickness initially increases in very early disease states—possibly reflecting neuroinflammation in response to amyloid pathology—and subsequently decreases with disease progression and onset of symptoms.^91^ Our results (Supplementary Figure 1a) provide longitudinal evidence that may be consistent with the downslope of such an inverted-U pattern, but these findings are somewhat tentative given that they were based on a post-hoc analysis. Furthermore, our concurrent analyses provide additional evidence that is consistent with this hypothesis because, at each wave (cross-sectionally), individuals who had already developed MCI tended to have lower thickness/volume signature scores compared to the robust-CN group at the same wave. Given these concurrent analyses were cross-sectional in nature and our statistical analysis showed no interaction between group and wave for either signature, we cannot draw firm conclusions about longitudinal changes in signature scores across waves from this set of analyses (Figure 2).

Prior evidence suggests that changes in MD may predate macrostructural atrophy as measured using conventional structural MRI techniques.^18^^,^^19^ MD may detect changes earlier and be more useful well before individuals convert to MCI; our finding that the MD signature was more predictive than the thickness/volume signature at average age 56 is in line with this prior evidence. MD may assess these early changes by directly measuring grey matter microstructure, or it may essentially be a more sensitive measure of cortical thinning due to possible bias by partial volume effects, such that increased contributions of signal from CSF due to subtle cortical thinning result in increased MD.^24^^,^^92^ However, we utilize a method to weight MD values based on the fraction of grey matter tissue in each sample, and our results suggest that partial volume effects may not be driving observed differences in MD. If MD were simply a reflection of partial volume effects due to cortical thinning, then lower MD signature scores would have to predict later MCI since higher cortical thickness signature scores predicted later MCI. We did not, however, observe such an inverse relationship. Rather, in our predictive analyses among only CN individuals, we found that both higher MD signature scores and greater thickness/volume signature scores at Wave 1 were associated with increased risk of incident MCI. This result thus suggests that the MD signature captured some unique information separate from the thickness/volume signature. In addition, we know from prior work from our group that variation in cortical and subcortical grey matter MD is partly influenced by genetic factors that are distinct from genetic factors influencing cortical thickness or subcortical volumes.^24^^,^^25^

Studies examining early changes in MD have reported mixed findings. Some studies demonstrate higher grey matter MD in presymptomatic individuals with autosomal dominant Alzheimer’s disease,^93^ while others have reported initial lower grey matter MD in CN individuals with evidence of Alzheimer’s disease pathology^89^ or in presymptomatic individuals with autosomal dominant Alzheimer’s disease^91^ that mirrors their findings of increased cortical thickness in early stages of Alzheimer’s disease. Montal et al.^89^ describe a biphasic model of changes in Alzheimer’s disease in which higher cortical thickness and lower MD occur in early preclinical Alzheimer’s disease stages, before reaching an inflection point at which cortical thickness decreases, MD increases, and clinical symptoms emerge. In our post-hoc longitudinal analyses examining the MD signature across age (Supplementary Figure 1b), we found MD signature scores trended higher (worse) across age for the CN-MCI group compared to the CN-stable group, though these supplemental findings are tentative. If the inflection point of changes in MD occurs earlier than the inflection point of changes in cortical thickness and, therefore, before the baseline age in our sample, it is possible that our results may be consistent with a biphasic pattern of changes. However, additional work is needed to examine the temporal relationship between cortical thickness and MD signatures. We also found that, among the subset of our sample comprising robust-CN and MCI groups, the correlation between the two Alzheimer’s disease signatures increased in magnitude from Wave 1 (*r* = −0.28) to Wave 2 (*r* = −0.50) to Wave 3 (*r* = −0.62), which suggests that the utility and optimal combination of signatures could change at different ages or disease states. Additional longitudinal studies are warranted to further elucidate how cortical thickness and MD may change across age and disease state, and to clarify processes underlying the MD differences.

Here, we note some limitations of the study. For our novel MD signature, we used ROIs and associated weights that were optimized to discriminate Alzheimer’s disease patients from controls based on cortical thickness and hippocampal volume, not MD. It is possible that an MD signature optimized to distinguish Alzheimer’s disease from control subjects may provide improved predictive ability over that observed here. Our results also suggest the potential value of developing a new MD signature similar to the way the thickness/volume signature was developed. It is possible that there are some differences in PBAD scores due to the fact that the BARACUS algorithm was developed on 3 T scanners, but PBAD scores in the present study were based on scans from study Wave 1, when imaging was conducted on 1.5 T scanners. However, evidence strongly suggests that the PBAD scores in the present study are valid. The brain age estimates were highly correlated with estimates from later study waves when 3 T scanners were employed. In addition, heritability estimates were very similar across waves and, as would be expected, brain age increased with increasing age in subsequent waves. In our supplemental analyses, inferences about within-subject change in signature scores across age are limited due to scanner differences across waves. However, signature scores were adjusted for scanner type and were *z*-scored at each wave, which may reduce the influence of scanner differences. Although we examined AD-PRSs, we were unable to confirm that MCI cases were Alzheimer’s disease-related based on Alzheimer’s disease biomarkers. Generalizability may be limited because the VETSA sample comprises only men and is largely white-non-Hispanic. However, the participants comprising a community-dwelling sample with average educational attainment and not being selected for elevated Alzheimer’s disease risk improves generalizability. As with most efforts to predict Alzheimer’s disease risk, our results were significant at the group level. Although this study represents a step in the direction of early prediction, the observed heterogeneity in individual signature scores across age (Supplementary Figure 1) underscores the need for additional investigation into individual change in Alzheimer’s disease signatures over time.

## Conclusions

An Alzheimer’s disease signature based on grey matter MD among CN adults who were only in their 50 s significantly improved prediction of progression to MCI over a follow-up period of 12 years. Grey matter MD signatures may serve as useful imaging biomarkers that are sensitive to Alzheimer’s disease-related changes as early as middle age. The results constitute a step towards early risk prediction in individuals much younger (by an average of approximately 20 years) than in most prior studies. Our findings are also in line with previous evidence suggesting that at early stages of Alzheimer’s disease, there may be an inverted-U pattern of cortical thickness. Taken together, these results suggest that the predictive utility of Alzheimer’s disease signatures from different modalities may change as a function of age and disease state. Future work may benefit from further exploration of the nature of these changes and from examining the predictive utility of MRI-based Alzheimer’s disease signatures in combination with biomarkers of Alzheimer’s disease pathology among middle-aged adults.

## Supplementary material

Supplementary material is available at *Brain Communications* online.

## Supplementary Material

fcab167_Supplementary_Data

## Abbreviations

ADNI =: Alzheimer’s Disease Neuroimaging Initiative
AD-PRS =: Alzheimer’s disease polygenic risk score
AUC =: area under the curve
CN =: cognitively normal
dMRI =: diffusion magnetic resonance imaging
MCI =: mild cognitive impairment
MD =: mean diffusivity
NODDI =: neurite density and orientation dispersion
OR =: odds ratio
PBAD =: predicted brain age difference
ROI =: region of interest
SMD =: standardized mean difference
VETSA =: Vietnam Era Twin Study of Aging

## References

[1] Vlassenko AG, McCue L, Jasielec MS, et al Imaging and cerebrospinal fluid biomarkers in early preclinical Alzheimer disease. Ann Neurol. 2016;80(3):379–387. 27398953 10.1002/ana.24719 PMC5016232

[2] Lerch JP, Pruessner JC, Zijdenbos A, Hampel H, Teipel SJ, Evans AC. Focal decline of cortical thickness in Alzheimer's disease identified by computational neuroanatomy. Cereb Cortex. 2005;15(7):995–1001. 15537673 10.1093/cercor/bhh200

[3] Du AT, Schuff N, Kramer JH, et al Different regional patterns of cortical thinning in Alzheimer's disease and frontotemporal dementia. Brain. 2006;130(4):1159–1166. 10.1093/brain/awm016 PMC1853284 17353226

[4] Singh V, Chertkow H, Lerch JP, Evans AC, Dorr AE, Kabani NJ. Spatial patterns of cortical thinning in mild cognitive impairment and Alzheimer's disease. Brain. 2006;129(Pt 11):2885–2893. 17008332 10.1093/brain/awl256

[5] Dickerson BC, Bakkour A, Salat DH, et al The cortical signature of Alzheimer's disease: Regionally specific cortical thinning relates to symptom severity in very mild to mild AD dementia and is detectable in asymptomatic amyloid-positive individuals. Cereb Cortex. 2009;19(3):497–510. 18632739 10.1093/cercor/bhn113 PMC2638813

[6] Sabuncu MR, Desikan RS, Sepulcre J, et al Alzheimer's Disease Neuroimaging Initiative. The dynamics of cortical and hippocampal atrophy in Alzheimer disease. Arch Neurol. 2011;68(8):1040–1048. 21825241 10.1001/archneurol.2011.167 PMC3248949

[7] Pettigrew C, Soldan A, Zhu Y, et al BIOCARD Research Team. Cortical thickness in relation to clinical symptom onset in preclinical AD. Neuroimage Clin. 2016;12:116–122. 27408796 10.1016/j.nicl.2016.06.010 PMC4932610

[8] Bakkour A, Morris JC, Dickerson BC. The cortical signature of prodromal AD: Regional thinning predicts mild AD dementia. Neurology. 2009;72(12):1048–1055. 19109536 10.1212/01.wnl.0000340981.97664.2f PMC2677470

[9] McEvoy LK, Fennema-Notestine C, Roddey JC, et al Alzheimer's Disease Neuroimaging Initiative. Alzheimer disease: Quantitative structural neuroimaging for detection and prediction of clinical and structural changes in mild cognitive impairment. Radiology. 2009;251(1):195–205. 19201945 10.1148/radiol.2511080924 PMC2663582

[10] Dickerson BC, Stoub TR, Shah RC, et al Alzheimer-signature MRI biomarker predicts AD dementia in cognitively normal adults. Neurology. 2011;76(16):1395–1402. 21490323 10.1212/WNL.0b013e3182166e96 PMC3087406

[11] McEvoy LK, Holland D, Hagler DJ, Fennema-Notestine C, Brewer JB, Dale AM., Alzheimer's Disease Neuroimaging Initiative. Mild cognitive impairment: Baseline and longitudinal structural MR imaging measures improve predictive prognosis. Radiology. 2011;259(3):834–843. 21471273 10.1148/radiol.11101975 PMC3099042

[12] Putcha D, Brickhouse M, O'Keefe K, et al Hippocampal hyperactivation associated with cortical thinning in Alzheimer's disease signature regions in non-demented elderly adults. J Neurosci. 2011;31(48):17680–17688. 22131428 10.1523/JNEUROSCI.4740-11.2011 PMC3289551

[13] Dickerson BC, Wolk DA, Alzheimer's Disease Neuroimaging Initiative. MRI cortical thickness biomarker predicts AD-like CSF and cognitive decline in normal adults. Neurology. 2012;78(2):84–90. 22189451 10.1212/WNL.0b013e31823efc6c PMC3466670

[14] Bakkour A, Morris JC, Wolk DA, Dickerson BC. The effects of aging and Alzheimer's disease on cerebral cortical anatomy: Specificity and differential relationships with cognition. Neuroimage. 2013;76:332–344. 23507382 10.1016/j.neuroimage.2013.02.059 PMC4098706

[15] Salat DH, Buckner RL, Snyder AZ, et al Thinning of the cerebral cortex in aging. Cereb Cortex. 2004;14(7):721–730. 15054051 10.1093/cercor/bhh032

[16] Racine AM, Brickhouse M, Wolk DA, Dickerson BC, Alzheimer's Disease Neuroimaging Initiative. Alzheimer's Disease Neuroimaging I. The personalized Alzheimer's disease cortical thickness index predicts likely pathology and clinical progression in mild cognitive impairment. Alzheimers Dement (Amst). 2018;10:301–310. 29780874 10.1016/j.dadm.2018.02.007 PMC5956936

[17] Vogt NM, Hunt JF, Adluru N, et al Cortical microstructural alterations in mild cognitive impairment and Alzheimer's disease dementia. Cereb Cortex. 2020;30(5):2948–2960. 31833550 10.1093/cercor/bhz286 PMC7197091

[18] Weston PS, Simpson IJ, Ryan NS, Ourselin S, Fox NC. Diffusion imaging changes in grey matter in Alzheimer's disease: A potential marker of early neurodegeneration. Alzheimers Res Ther. 2015;7(1):47. 26136857 10.1186/s13195-015-0132-3 PMC4487800

[19] Kantarci K. Magnetic resonance markers for early diagnosis and progression of Alzheimer's disease. Expert Rev Neurother. 2005;5(5):663–670. 16162090 10.1586/14737175.5.5.663

[20] Kantarci K, Jack CR Jr, Xu YC, et al Regional diffusivity of water in mild cognitive impairment and Alzheimer’s disease. Radiology. 2001;219(1):101–107. 11274543 10.1148/radiology.219.1.r01ap14101 PMC2771587

[21] Fellgiebel A, Wille P, Muller MJ, et al Ultrastructural hippocampal and white matter alterations in mild cognitive impairment: A diffusion tensor imaging study. Dement Geriatr Cogn Disord. 2004;18(1):101–108. 15087585 10.1159/000077817

[22] Ray KM, Wang H, Chu Y, et al Mild cognitive impairment: Apparent diffusion coefficient in regional gray matter and white matter structures. Radiology. 2006;241(1):197–205. 16990677 10.1148/radiol.2411051051

[23] Scola E, Bozzali M, Agosta F, et al A diffusion tensor MRI study of patients with MCI and AD with a 2-year clinical follow-up. J Neurol Neurosurg Psychiatry. 2010;81(7):798–805. 20392973 10.1136/jnnp.2009.189639

[24] Elman JA, Panizzon MS, Hagler DJ Jr, et al Genetic and environmental influences on cortical mean diffusivity. Neuroimage. 2017;146:90–99. 27864081 10.1016/j.neuroimage.2016.11.032 PMC5322245

[25] Gillespie NA, Neale MC, Hagler DJ Jr, et al Genetic and environmental influences on mean diffusivity and volume in subcortical brain regions. Hum Brain Mapp. 2017;38(5):2589–2598. 28240386 10.1002/hbm.23544 PMC5810123

[26] Brookmeyer R, Gray S, Kawas C. Projections of Alzheimer’s disease in the United States and the public health impact of delaying disease onset. Am J Public Health. 1998;88(9):1337–1342. 9736873 10.2105/ajph.88.9.1337 PMC1509089

[27] Brookmeyer R, Johnson E, Ziegler-Graham K, Arrighi HM. Forecasting the global burden of Alzheimer's disease. Alzheimers Dement. 2007;3(3):186–191. 19595937 10.1016/j.jalz.2007.04.381

[28] Hebert LE, Weuve J, Scherr PA, Evans DA. Alzheimer disease in the United States (2010-2050) estimated using the 2010 census. Neurology. 2013;80(19):1778–1783. 23390181 10.1212/WNL.0b013e31828726f5 PMC3719424

[29] Riedel BC, Thompson PM, Brinton RD. Age, APOE and sex: Triad of risk of Alzheimer's disease. J Steroid Biochem Mol Biol. 2016;160:134–147. 26969397 10.1016/j.jsbmb.2016.03.012 PMC4905558

[30] Schmoldt A, Benthe HF, Haberland G. An Alzheimer's disease genetic risk score predicts longitudinal thinning of hippocampal complex subregions in healthy older adults. eNeuro. 1975;24(17):1639–1641. 10.1523/ENEURO.0098-16.2016 PMC4945997 27482534

[31] Lupton MK, Strike L, Hansell NK, et al Alzheimer's Disease Neuroimaging Initiative. The effect of increased genetic risk for Alzheimer's disease on hippocampal and amygdala volume. Neurobiol Aging. 2016;40:68–77. 26973105 10.1016/j.neurobiolaging.2015.12.023 PMC4883003

[32] Mormino EC, Sperling RA, Holmes AJ, et al For the Alzheimer's Disease Neuroimaging Initiative. Polygenic risk of Alzheimer disease is associated with early- and late-life processes. Neurology. 2016;87(5):481–488. 27385740 10.1212/WNL.0000000000002922 PMC4970660

[33] Logue MW, Panizzon MS, Elman JA, et al Use of an Alzheimer's disease polygenic risk score to identify mild cognitive impairment in adults in their 50s. Mol Psychiatry. 2019;24(3):421–430. 29487403 10.1038/s41380-018-0030-8 PMC6110977

[34] Kremen WS, Thompson-Brenner H, Leung YM, et al Genes, environment, and time: The Vietnam era twin study of aging (VETSA). Twin Res Human Genet. 2006;9(6):1009–1022. 17254445 10.1375/183242706779462750

[35] Kremen WS, Franz CE, Lyons MJ. VETSA: The Vietnam Era Twin Study of Aging. Twin Res Hum Genet. 2013;16(1):399–402. 23110957 10.1017/thg.2012.86 PMC3780387

[36] Kremen WS, Franz CE, Lyons MJ. Current status of the Vietnam Era Twin Study of Aging (VETSA). Twin Res Hum Genet. 2019;22(6):783–787. 31933447 10.1017/thg.2019.125

[37] Heron M, Hoyert DL, Murphy SL, Xu J, Kochanek KD, Tejada-Vera B. Health characteristics of adults aged 55 years and over: United States, 2004–2007. Natl Health Stat Rep. 2009;57(14):1–31. 19697804

[38] Kremen WS, Jak AJ, Panizzon MS, et al Early identification and heritability of mild cognitive impairment. Int J Epidemiol. 2014;43(2):600–610. 24370560 10.1093/ije/dyt242 PMC3997374

[39] Bondi MW, Jak AJ, Delano-Wood L, Jacobson MW, Delis DC, Salmon DP. Neuropsychological contributions to the early identification of Alzheimer's disease. Neuropsychol Rev. 2008;18(1):73–90. 18347989 10.1007/s11065-008-9054-1 PMC2882236

[40] Jak AJ, Bondi MW, Delano-Wood L, et al Quantification of five neuropsychological approaches to defining mild cognitive impairment. Am J Geriatr Psychiatry. 2009;17(5):368–375. 19390294 10.1097/JGP.0b013e31819431d5 PMC2743175

[41] Bondi MW, Edmonds EC, Jak AJ, et al Neuropsychological criteria for mild cognitive impairment improves diagnostic precision, biomarker associations, and progression rates. J Alzheimers Dis. 2014;42(1):275–289. 24844687 10.3233/JAD-140276 PMC4133291

[42] Granholm EL, Panizzon MS, Elman JA, et al Pupillary responses as a biomarker of early risk for Alzheimer's disease. J Alzheimers Dis. 2017;56(4):1419–1428. 28157098 10.3233/JAD-161078 PMC5808562

[43] Lyons MJ, Panizzon MS, Liu W, et al A longitudinal twin study of general cognitive ability over four decades. Dev Psychol. 2017;53(6):1170–1177. 28358535 10.1037/dev0000303 PMC5474938

[44] Ronnlund M, Nyberg L, Backman L, Nilsson LG. Stability, growth, and decline in adult life span development of declarative memory: Cross-sectional and longitudinal data from a population-based study. Psychol Aging. 2005;20(1):3–18. 15769210 10.1037/0882-7974.20.1.3

[45] Elman JA, Jak AJ, Panizzon MS, et al Underdiagnosis of mild cognitive impairment: A consequence of ignoring practice effects. Alzheimers Dement (Amst). 2018;10:372–381. 30003138 10.1016/j.dadm.2018.04.003 PMC6039708

[46] Fischl B, Salat DH, Busa E, et al Whole brain segmentation: Automated labeling of neuroanatomical structures in the human brain. Neuron. 2002;33(3):341–355. 11832223 10.1016/s0896-6273(02)00569-x

[47] Fischl B, van der Kouwe A, Destrieux C, et al Automatically parcellating the human cerebral cortex. Cereb Cortex. 2004;14(1):11–22. 14654453 10.1093/cercor/bhg087

[48] Dale AM, Sereno MI. Improved localizadon of cortical activity by combining EEG and MEG with MRI cortical surface reconstruction: A linear approach. J Cogn Neurosci. 1993;5(2):162–176. 23972151 10.1162/jocn.1993.5.2.162

[49] Dale AM, Fischl B, Sereno MI. Cortical surface-based analysis. I. Segmentation and surface reconstruction. Neuroimage. 1999;9(2):179–194. 9931268 10.1006/nimg.1998.0395

[50] Kremen WS, Prom-Wormley E, Panizzon MS, et al Genetic and environmental influences on the size of specific brain regions in midlife: The VETSA MRI study. Neuroimage. 2010;49(2):1213–1223. 19786105 10.1016/j.neuroimage.2009.09.043 PMC3397915

[51] McEvoy LK, Fennema-Notestine C, Eyler LT, et al Hypertension-related alterations in white matter microstructure detectable in middle age. Hypertension. 2015;66(2):317–323. 26056337 10.1161/HYPERTENSIONAHA.115.05336 PMC4499000

[52] Jovicich J, Czanner S, Greve D, et al Reliability in multi-site structural MRI studies: Effects of gradient non-linearity correction on phantom and human data. Neuroimage. 2006;30(2):436–443. 16300968 10.1016/j.neuroimage.2005.09.046

[53] Sled JG, Zijdenbos AP, Evans AC. A nonparametric method for automatic correction of intensity nonuniformity in MRI data. IEEE Trans Med Imaging. 1998;17(1):87–97. 9617910 10.1109/42.668698

[54] Desikan RS, Segonne F, Fischl B, et al An automated labeling system for subdividing the human cerebral cortex on MRI scans into gyral based regions of interest. Neuroimage. 2006;31(3):968–980. 16530430 10.1016/j.neuroimage.2006.01.021

[55] Holland D, Kuperman JM, Dale AM. Efficient correction of inhomogeneous static magnetic field-induced distortion in Echo Planar Imaging. Neuroimage. 2010;50(1):175–183. 19944768 10.1016/j.neuroimage.2009.11.044 PMC2819607

[56] Chang H, Fitzpatrick JM. A technique for accurate magnetic resonance imaging in the presence of field inhomogeneities. IEEE Trans Med Imaging. 1992;11(3):319–329. 18222873 10.1109/42.158935

[57] Morgan PS, Bowtell RW, McIntyre DJ, Worthington BS. Correction of spatial distortion in EPI due to inhomogeneous static magnetic fields using the reversed gradient method. J Magn Reson Imaging. 2004;19(4):499–507. 15065175 10.1002/jmri.20032

[58] Zhuang J, Hrabe J, Kangarlu A, et al Correction of eddy-current distortions in diffusion tensor images using the known directions and strengths of diffusion gradients. J Magn Reson Imaging. 2006;24(5):1188–1193. 17024663 10.1002/jmri.20727 PMC2364728

[59] Hagler DJ Jr, Ahmadi ME, Kuperman J, et al Automated white-matter tractography using a probabilistic diffusion tensor atlas: Application to temporal lobe epilepsy. Hum Brain Mapp. 2009;30(5):1535–1547. 18671230 10.1002/hbm.20619 PMC2754725

[60] Leemans A, Jones DK. The B-matrix must be rotated when correcting for subject motion in DTI data. Magn Reson Med. 2009;61(6):1336–1349. 19319973 10.1002/mrm.21890

[61] Wells WM, Viola P, Atsumi H, Nakajima S, Kikinis R. Multi-modal volume registration by maximization of mutual information. Med Image Anal. 1996;1(1):35–51. 9873920 10.1016/s1361-8415(01)80004-9

[62] Basser PJ, Mattiello J, LeBihan D. MR diffusion tensor spectroscopy and imaging. Biophys J. 1994;66(1):259–267. 8130344 10.1016/S0006-3495(94)80775-1 PMC1275686

[63] Pierpaoli C, Jezzard P, Basser PJ, Barnett A, Di Chiro G. Diffusion tensor MR imaging of the human brain. Radiology. 1996;201(3):637–648. 8939209 10.1148/radiology.201.3.8939209

[64] Le Bihan D, Mangin JF, Poupon C, et al Diffusion tensor imaging: Concepts and applications. J Magn Reson Imaging. 2001;13(4):534–546. 11276097 10.1002/jmri.1076

[65] Beaton AE, Tukey JW. The fitting of power series, meaning polynomials, illustrated on band-spectroscopic data. Technometrics. 1974;16(2):147–185.

[66] Black MJ, Sapiro G, Marimont DH, Heeger D. Robust anisotropic diffusion. IEEE Trans Image Process. 1998;7(3):421–432. 18276262 10.1109/83.661192

[67] Hsu CC, Wu MT, Lee C. Robust image registration for functional magnetic resonance imaging of the brain. Med Biol Eng Comput. 2001;39(5):517–524. 11712647 10.1007/BF02345141

[68] Samaille T, Fillon L, Cuingnet R, et al Contrast-based fully automatic segmentation of white matter hyperintensities: Method and validation. PLoS One. 2012;7(11):e48953. 23152828 10.1371/journal.pone.0048953 PMC3495958

[69] Lambert JC, Ibrahim-Verbaas CA, Harold D, et al European Alzheimer's Disease Initiative (EADI). Meta-analysis of 74,046 individuals identifies 11 new susceptibility loci for Alzheimer's disease. Nat Genet. 2013;45(12):1452–1458. 24162737 10.1038/ng.2802 PMC3896259

[70] Martin AR, Kanai M, Kamatani Y, Okada Y, Neale BM, Daly MJ. Clinical use of current polygenic risk scores may exacerbate health disparities. Nat Genet. 2019;51(4):584–591. 30926966 10.1038/s41588-019-0379-x PMC6563838

[71] Liem F, Varoquaux G, Kynast J, et al Predicting brain-age from multimodal imaging data captures cognitive impairment. Neuroimage. 2017;148:179–188. 27890805 10.1016/j.neuroimage.2016.11.005

[72] Hatton SN, Franz CE, Elman JA, et al Negative fateful life events in midlife and advanced predicted brain aging. Neurobiol Aging. 2018;67:1–9. 29609076 10.1016/j.neurobiolaging.2018.03.004 PMC5955847

[73] Cohen J. A power primer. Psychol Bull. 1992;112(1):155–159. 19565683 10.1037//0033-2909.112.1.155

[74] Moore CS, Grant MD, Zink TA, et al Erectile dysfunction, vascular risk, and cognitive performance in late middle age. Psychol Aging. 2014;29(1):163–172. 24660805 10.1037/a0035463 PMC4850828

[75] Youden WJ. Index for rating diagnostic tests. Cancer. 1950;3(1):32–35. 15405679 10.1002/1097-0142(1950)3:1<32::aid-cncr2820030106>3.0.co;2-3

[76] Benjamini Y, Hochberg Y. Controlling the false discovery rate: A practical and powerful approach to multiple testing. J R Stat Soc. 1995;57:289–300.

[77] Robin X, Turck N, Hainard A, et al pROC: An open-source package for R and S+ to analyze and compare ROC curves. BMC Bioinformatics. 2011;12:77. 21414208 10.1186/1471-2105-12-77 PMC3068975

[78] Bates D, Mächler M, Bolker B, Walker S. Fitting linear mixed-effects models using lme4. J Stat Softw. 2015;67(1):1–48

[79] R Core Team (2020). R: A language and environment for statistical computing. Vienna, Austria: R Foundation for Statistical Computing. https://www.R-project.org/.

[80] Mowinckel AM, Vidal-Pieiro D. (2019). Visualisation of Brain Statistics with R-packages ggseg and ggseg3d. arXiv:1912.08200.

[81] Steenland K, Zhao L, Goldstein F, Cellar J, Lah J, for the Alzheimer's Disease Neuroimaging Initiative. Biomarkers for predicting cognitive decline in those with normal cognition. J Alzheimers Dis. 2014;40(3):587–594. 24496071 10.3233/JAD-2014-131343 PMC4462517

[82] Albert M, Zhu Y, Moghekar A, et al Predicting progression from normal cognition to mild cognitive impairment for individuals at 5 years. Brain. 2018;141(3):877–887. 29365053 10.1093/brain/awx365 PMC5837651

[83] Vos SJB, Xiong C, Visser PJ, et al Preclinical Alzheimer's disease and its outcome: A longitudinal cohort study. Lancet Neurol. 2013;12(10):957–965. 24012374 10.1016/S1474-4422(13)70194-7 PMC3904678

[84] Roberts RO, Aakre JA, Kremers WK, et al Prevalence and outcomes of amyloid positivity among persons without dementia in a longitudinal, population-based setting. JAMA Neurol. 2018;75(8):970–979. 29710225 10.1001/jamaneurol.2018.0629 PMC6142936

[85] van Maurik IS, Slot RER, Verfaillie SCJ, et al Alzheimer’s Disease Neuroimaging Initiative. Personalized risk for clinical progression in cognitively normal subjects-the ABIDE project. Alzheimers Res Ther. 2019;11(1):33. 30987684 10.1186/s13195-019-0487-y PMC6466790

[86] Fani L, Ahmad S, Ikram MK, et al Plasma tau, neurofilament light chain and amyloid-beta levels and risk of dementia; a population-based cohort study. Brain. 2021;17 (3):446– 1232. 10.1002/alz.12212 PMC8048997 33215849

[87] van der Kall LM, Truong T, Burnham SC, et al Association of beta-amyloid level, clinical progression, and longitudinal cognitive change in normal older individuals. Neurology. 2021;96(5):e662–e670. 33184233 10.1212/WNL.0000000000011222 PMC7884996

[88] Verberk IMW, Laarhuis MB, van den Bosch KA, et al Serum markers glial fibrillary acidic protein and neurofilament light for prognosis and monitoring in cognitively normal older people: A prospective memory clinic-based cohort study. Lancet Healthy Longev. 2021;2(2):e87–e95. 10.1016/S2666-7568(20)30061-1 36098162

[89] Montal V, Vilaplana E, Alcolea D, et al Cortical microstructural changes along the Alzheimer's disease continuum. Alzheimers Dement. 2018;14(3):340–351. 29080407 10.1016/j.jalz.2017.09.013

[90] Batzu L, Westman E, Pereira JB, Alzheimer's Disease Neuroimaging Initiative. Cerebrospinal fluid progranulin is associated with increased cortical thickness in early stages of Alzheimer's disease. Neurobiol Aging. 2020;88:61–70. 31980280 10.1016/j.neurobiolaging.2019.12.012

[91] Fortea J, Sala-Llonch R, Bartres-Faz D, et al Increased cortical thickness and caudate volume precede atrophy in PSEN1 mutation carriers. J Alzheimers Dis. 2010;22(3):909–922. 20858974 10.3233/JAD-2010-100678

[92] Koo BB, Hua N, Choi CH, Ronen I, Lee JM, Kim DS. A framework to analyze partial volume effect on gray matter mean diffusivity measurements. Neuroimage. 2009;44(1):136–144. 18775785 10.1016/j.neuroimage.2008.07.064

[93] Weston PSJ, Poole T, Nicholas JM, et al Measuring cortical mean diffusivity to assess early microstructural cortical change in presymptomatic familial Alzheimer's disease. Alzheimers Res Ther. 2020;12(1):112. 32943095 10.1186/s13195-020-00679-2 PMC7499910

