# Supplementary material for: 12-year prediction of mild cognitive impairment aided by Alzheimer’s brain signatures at mean age 56
Source: Brain Commun. 2021 Jul 23;3(3):fcab167. doi: 10.1093/braincomms/fcab167 (PMC8361427; doi:10.1093/braincomms/fcab167)
Supplement: fcab167_Supplementary_Data [file fcab167_supplementary_data.docx]

| **Supplementary Materials**  **Supplementary Figure 1a**. Cortical thickness/volume signature across age for participants included in predictive analyses (*n* = 169).  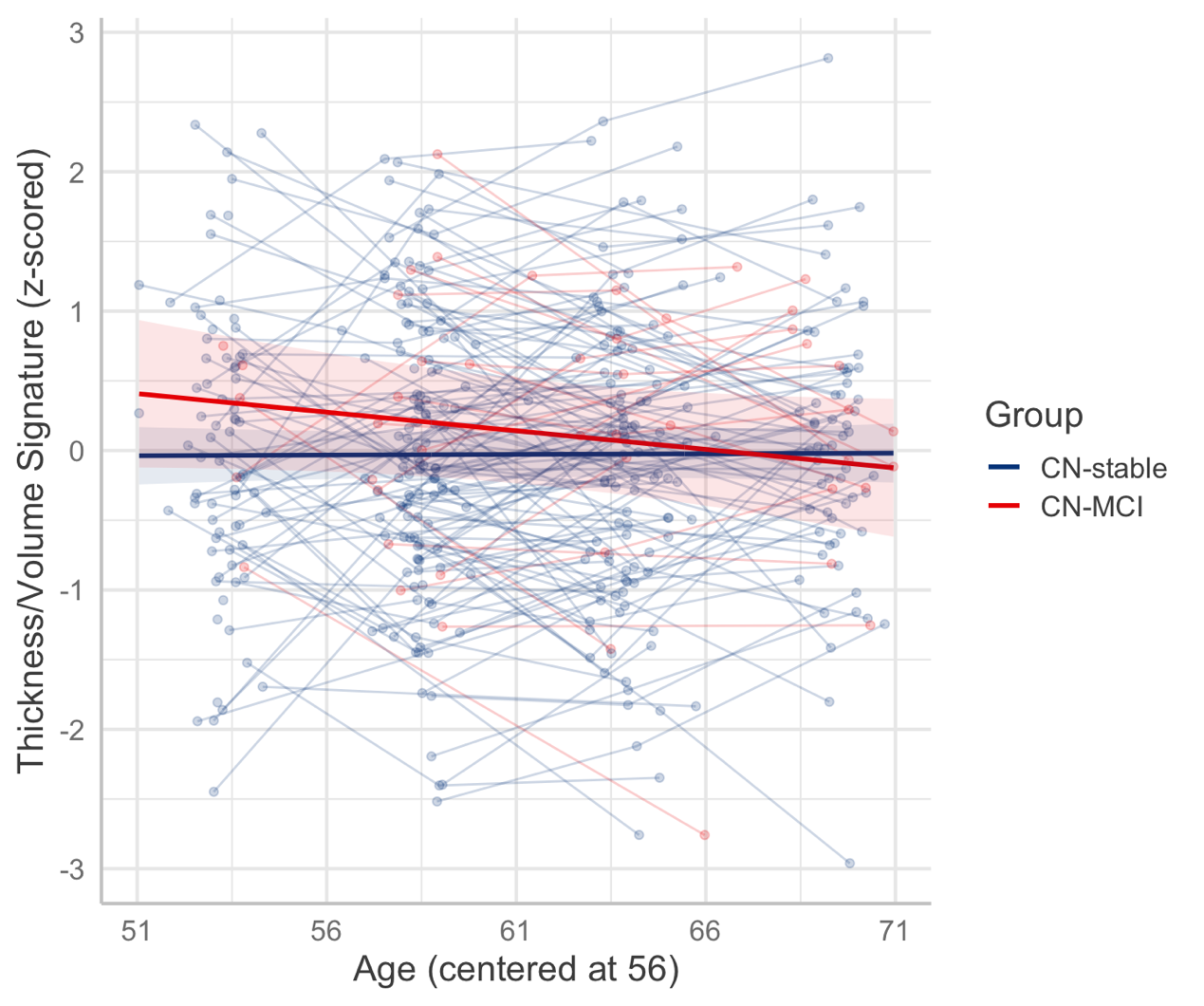 |
| --- |

Abbreviations: CN-stable, remained cognitively normal across waves; CN-MCI, cognitively normal and progressed to mild cognitive impairment.

All were CN at wave 1. Model predictions from a mixed model that accounts for non-independence of twins and repeated measurement across waves. Predictors include age (*t*_286.13_ = 0.14; *p* = 0.892), group (CN-stable or CN-MCI; *t*_252.67_ = 1.38; *p* = 0.170), and allows for a linear interaction between age and group (*t*_288.19_ = -1.45; *p* = 0.148). A quadratic term for age was tested but was not significant and resulted in poor model fit [χ^2^(2) = 0.30; *p* = 0.861]. Signature scores were z-scored within each wave. Note: heterogeneity in individual trajectories across age may be influenced by scanner differences across waves (1.5 T scanner at wave 1, 3T scanners at waves 2 and 3), thus inferences about within-subject longitudinal change are limited.

**Supplementary Figure 1b.** MD signature across age for participants included in predictive analyses (*n* = 169).


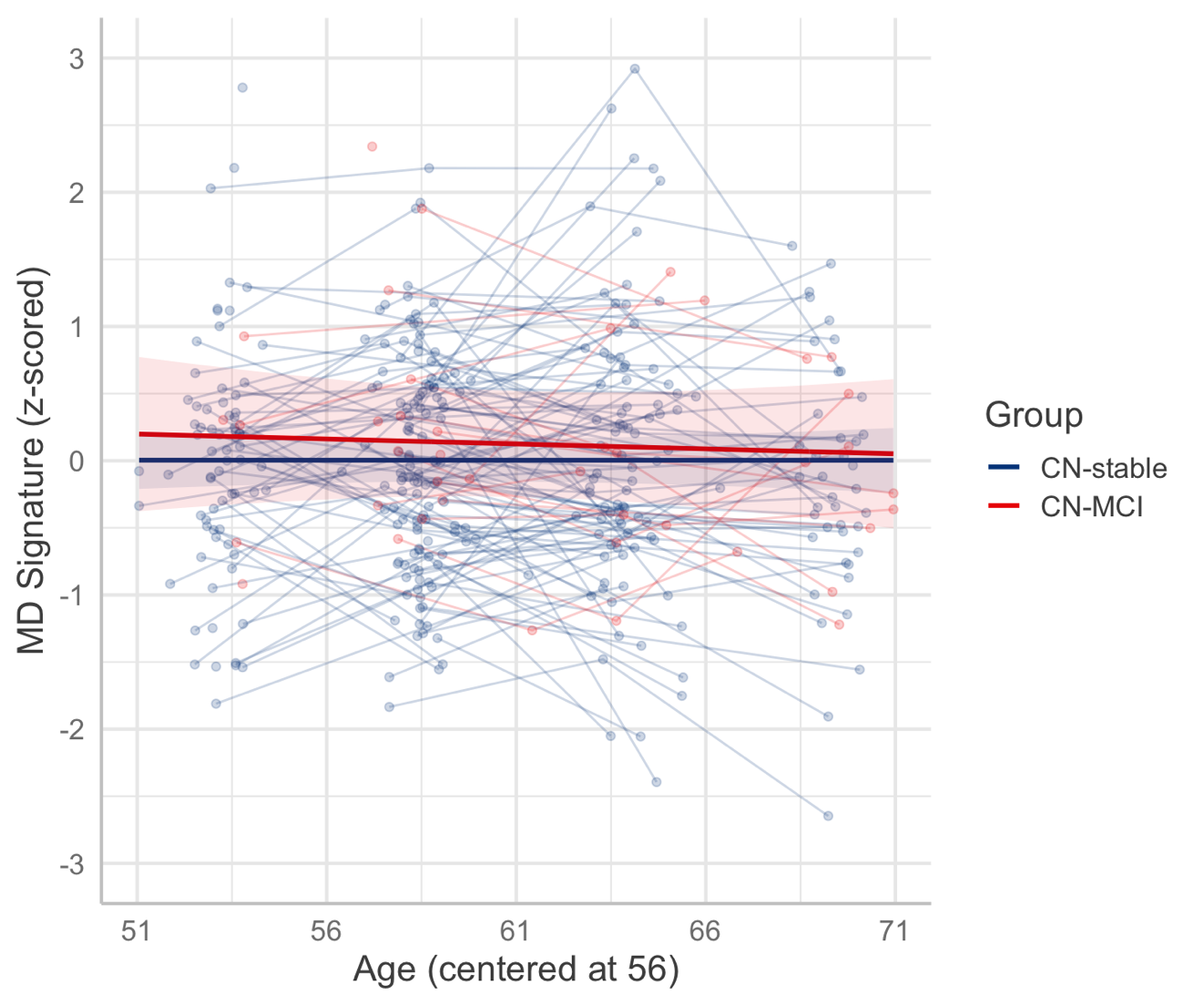


Abbreviations: MD, mean diffusivity; CN-stable, remained cognitively normal across waves; CN-MCI, cognitively normal and progressed to mild cognitive impairment.

All were CN at wave 1. Model predictions from a mixed model that accounts for non-independence of twins and repeated measurement across waves. Predictors include age (*t*_240.40_ = -0.004; *p* = 0.996), group (CN-stable or CN-MCI; *t*_250.50_ = 0.661; *p* = 0.509), and allows for a linear interaction between age and group (*t*_236.00_ = -0.33; *p* = 0.742). A quadratic term for age was tested but was not significant and resulted in poor model fit [χ^2^(2) = 0.82; *p* = 0.663]. Signature scores were z-scored within each wave. Note: heterogeneity in individual trajectories across age may be influenced by scanner differences across waves (1.5 T scanner at wave 1, 3T scanners at waves 2 and 3), thus inferences about within-subject longitudinal change are limited.

| **Supplementary Table 1**. Predicting 12-year progression to MCI: ROC results. | | | | |  | |  | |  |
| --- | --- | --- | --- | --- | --- | --- | --- | --- | --- |
| Model | AUC | Sensitivity | Specificity | Accuracy | | PPV | | NPV | |
| Age | 0.64 | 0.95 | 0.40 | 0.46 | | 0.17 | | 0.98 | |
| ADPRS | 0.72 | 0.75 | 0.62 | 0.64 | | 0.21 | | 0.95 | |
| Age + ADPRS | 0.74 | 0.80 | 0.65 | 0.67 | | 0.24 | | 0.96 | |
| Thickness/Vol | 0.56 | 0.75 | 0.43 | 0.47 | | 0.15 | | 0.93 | |
| Thickness/Vol + ADPRS | 0.74 | 0.75 | 0.70 | 0.70 | | 0.25 | | 0.95 | |
| Thickness/Vol + Age | 0.62 | 0.95 | 0.29 | 0.37 | | 0.15 | | 0.98 | |
| Thickness/Vol + Age + ADPRS | 0.80 | 0.60 | 0.90 | 0.86 | | 0.43 | | 0.94 | |
| PBAD-Adjusted Thickness/Vol + Age + ADPRS | 0.81 | 0.65 | 0.89 | 0.86 | | 0.43 | | 0.95 | |
| MD | 0.57 | 0.95 | 0.24 | 0.33 | | 0.14 | | 0.97 | |
| MD + ADPRS | 0.69 | 0.45 | 0.87 | 0.82 | | 0.32 | | 0.92 | |
| MD + Age | 0.66 | 0.75 | 0.56 | 0.58 | | 0.19 | | 0.94 | |
| MD + Age + ADPRS | 0.83 | 0.85 | 0.78 | 0.79 | | 0.34 | | 0.97 | |
| PBAD-Adjusted MD + Age + ADPRS | 0.81 | 0.90 | 0.68 | 0.71 | | 0.28 | | 0.98 | |
| Thickness/Vol + MD | 0.60 | 0.60 | 0.64 | 0.64 | | 0.18 | | 0.92 | |
| Thickness/Vol + MD + ADPRS | 0.74 | 0.70 | 0.71 | 0.71 | | 0.25 | | 0.95 | |
| Thickness/Vol + MD + Age | 0.64 | 0.50 | 0.73 | 0.70 | | 0.20 | | 0.92 | |
| Thickness/Vol + MD + Age + ADPRS | 0.83 | 0.75 | 0.82 | 0.81 | | 0.36 | | 0.96 | |
| PBAD-Adjusted Signatures + Age + ADPRS | 0.82 | 0.70 | 0.85 | 0.83 | | 0.39 | | 0.95 | |
| Age + *APOE-*ε4 | 0.55 | 0.30 | 0.86 | 0.79 | | 0.22 | | 0.90 | |
| Thickness/Vol + Age + *APOE-*ε4 | 0.67 | 0.60 | 0.72 | 0.70 | | 0.22 | | 0.93 | |
| MD + Age + *APOE-*ε4 | 0.67 | 0.80 | 0.52 | 0.56 | | 0.18 | | 0.95 | |
| Thickness/Vol + MD + Age + *APOE-*ε4 | 0.70 | 0.45 | 0.89 | 0.84 | | 0.36 | | 0.92 | |
| PBAD-Adjusted Signatures + Age + *APOE-*ε4 | 0.70 | 0.90 | 0.42 | 0.48 | | 0.17 | | 0.97 | |
| Abbreviations: MCI, mild cognitive impairment; ROC, receiver operating characteristics; AUC, area under the curve; PPV, positive predictive value; NPV, negative predictive value; ADPRS, Alzheimer’s disease polygenic risk score; Thickness /Vol: cortical thickness/volume signature; PBAD, predicted brain age difference; MD, mean diffusivity signature.  Optimal threshold was selected for high and balanced sensitivity and specificity (Youden method). | | | | | | | | |  |

***Sample sizes across waves in concurrent analyses***

The VETSA sample comprises participants involved in multiple waves, some of whom entered the study at different waves. For the thickness/volume signature, 246 participants had data for only one wave, 183 had data for two waves, and 132 had data for all three waves (for a total of 561 participants with thickness/volume signatures). For the MD signature, 262 participants had data for only one wave, 170 had data for two waves, and 65 had data for all three waves (for a total of 497 participants with MD signatures). Three participants that had data for MD signatures did not have useable data for thickness/volume signatures at any wave. *APOE-*ε4 status was not available for 43 of the 564 total participants and AD-PRS data were not available for 95 of the 564 total participants.
